# Liver Transplantation in the Context of Acute-On-Chronic Liver Failure (ACLF): Where Do We Stand in 2025?

**DOI:** 10.3389/ti.2025.14752

**Published:** 2025-08-05

**Authors:** Sébastien L’Hermite, Valentin Coirier, Florent Artru

**Affiliations:** ^1^Service des maladies du foie, hôpital Pontchaillou, CHU Rennes, Rennes Liver Failure Group RELIEF, Rennes, France; ^2^ Université de Rennes et institut NuMeCan Inserm U1241, Rennes, France; ^3^Service de Médecine Intensive Réanimation, hôpital Pontchaillou, CHU Rennes, Rennes Liver Failure Group RELIEF, Rennes, France

**Keywords:** cirrhosis, acute-on-chronic liver failure (ACLF), liver transplantation, liver transplantation window, organ failure

## Abstract

Acute-on-chronic liver failure (ACLF) is a critical condition that arises in the context of advanced liver disease, marked by rapid liver function deterioration and associated multi-organ failure. This syndrome is associated with a major short-term mortality risk, requiring aggressive and specialized clinical care. Despite ongoing efforts, effective therapeutic options for ACLF are lacking, with liver transplantation (LT) considered the only life-saving intervention, yielding acceptable outcomes in carefully selected patients. However, the place of LT for ACLF remains a matter of debate, given the high prevalence of the syndrome, the sickness of liver transplant candidates, the persistent shortage of available liver grafts, and the increasing number of indications to LT. This review aims to provide a comprehensive analysis of the role of LT in ACLF, evaluating current evidence on patient selection, optimal timing for transplantation, and ongoing debates surrounding this practice, specifically the rationale for prioritizing graft allocation for this indication. Furthermore, we will explore global management strategies for ACLF, focusing on bridging patients to LT and improving survival outcomes. Through this review, we seek to enhance understanding of the evolving role of LT in ACLF and offer insights into future directions for clinical practice and research in this critical area.

## Introduction

In patients with chronic liver disease, an acute insult—whether intrahepatic or extrahepatic—can precipitate both hepatic and extrahepatic organ failures, a syndrome now recognized as acute-on-chronic liver failure (ACLF). ACLF is characterized by hepatic failure occurring in the setting of chronic liver disease, combined with extrahepatic organ failures, leading to a high risk of short-term mortality [1]. Among patients hospitalized for acutely decompensated cirrhosis, ACLF was present in 22.6% at admission and developed during hospitalization in an additional 8.3% [2]. The European Association for the Study of the Liver–Chronic Liver Failure (EASL-CLIF) Consortium defines six organ failures (OFs) relevant for ACLF diagnosis: liver, kidney, and brain function, along with coagulation, circulation, and respiration. Dysfunction and failure of organ systems are based on thresholds merged in the CLIF-C OF score [3]. As per EASL-CLIF definition, ACLF is classified into three grades (ACLF-1 to ACLF-3) based on the number of OFs, with higher grades correlating with increased mortality. Other definitions, such as that of the Asian Pacific Association for the Study of the Liver (APASL), differ regarding the underlying stage of liver disease, prior episodes of decompensation, the severity of OFs, and the inclusion of extrahepatic failures [4]. Despite these disparities, all definitions converge on the poor prognosis associated with ACLF [5]. According to the European definition, patients with at least three OFs (ACLF-3) face a 28-day mortality rate exceeding 80% [6]. According to the APASL-ACLF Research Consortium (AARC) scoring system patients with an AARC grade III (corresponding to 3 extrahepatic organ failures), face a 28-day mortality risk of 85.9% [4].

ACLF also has a distinct pathophysiology compared to decompensated cirrhosis, with intense systemic inflammation being the cornerstone of its pathogenesis. Studies have demonstrated a direct correlation between systemic inflammation and ACLF severity: the greater the inflammatory response, the higher the number of OFs at diagnosis and the greater the short-term mortality [7]. In Western countries, bacterial infections and alcohol-associated hepatitis (AH) are the most common precipitating factors [8], similarly alcohol consumption, infections and hemorrhage were the most frequent factors in Latin America [9]. Systemic inflammation is primarily driven by hepatic cell death and inflammatory processes, which enhance bacterial translocation from the gut [10]. This translocation triggers a proinflammatory response, followed by a compensatory systemic anti-inflammatory reaction, leading to immune suppression and an increased risk of infections. This inflammatory state is marked by significantly elevated plasma levels of proinflammatory cytokines (IL-6 and TNF-α), chemokines, adhesion molecules, soluble markers of macrophage activation, and circulating white blood cells [10].

In this review, we provide an in-depth analysis of existing evidence, focusing on LT outcomes and predictive mortality factors in ACLF, patient prioritization on the LT waiting list, and future perspectives on LT for ACLF.

## Outcomes of Liver Transplantation in ACLF

### Short-Term Outcomes of Deceased-Donor-LT (DDLT)

In this review, we will focus exclusively on studies that included patients with ACLF at the time of LT, as defined by either the EASL or APASL criteria as most studies investigation LT outcome in ACLF setting were based on these definitions (Table 1).

**TABLE 1:** Outcomes of studies evaluating liver transplantation for ACLF.

Study	Study period	ACLF grades 1; 2; 3* (n)	1-year post-LT survival for ACLF grades 1; 2; 3*	Long-term post-LT survival for ACLF grades 1; 2; 3*
Deceased-donor liver transplantation				
Kwon et al. [11]	2008–2019	102; 129; 140	ACLF grade 3: 67.9%	5-year survival : 57.6%
Artru et al. [32]	2008–2014	ACLF grade 3 : 73	NA	5-year survival 76.4%; 69.7%; 72.6%; 10-year survival 58.6%; 58.3%; 56.8%;
Bernal et al. [30]	2021–2023	ACLF grade 3 : 42	ACLF grade 3 : 77%	NA
Alukal et al. [29]	2005–2021	ACLF grade 3 : 4806	ACLF grade 3: 86.2%	NA
Hernaez et al. [43]	2014–2019	0; 237; 284	84.4% for grade 2; 76.4% for grade 3	NA
Zhu et al. [12]	2018–2020	75; 64; 73	93.3%; 73.4%; 60.3%	NA
Xia et al. [13]	2015–2021	18; 97; 47	83.0%; 83.2%; 69.8%	3-year survival 83.0%; 80.3%; 69.8%
Sundaram et al. [28]	2018–2019	61; 74; 77	88.5%; 87.8%; 85.7%	NA
Cervantes-Alvarez et al. [14]	2015–2019	40; 33; 22	87.5%; 97.0%; 90.9%	6-year survival 80.0%; 93.9%; 77.3%
Artzner et al. [39]	2018–2019	ACLF grade 3 : 98	ACLF grade 3: 79%	NA
Goosmann et al. [15]	2009–2014	All grades : 98	NA	5-year survival 55.1%
Belli et al. [27]	2018–2019	58; 78; 98	88.6% for grade 1; 78.9% for grade 3	NA
Sundaram et al. [26]	2004–2017	ACLF grade 3 : 2744	ACLF grade 3: 82%	NA
Artzner et al. [47]	2007–2017	ACLF grade 3 : 152	ACLF grade 3: 67.1%	NA
Agbim et al. [16]	2006–2013	50; 32; 19	86%; 81%; 74%	NA
Sundaram et al. [26]	2004–2017	8757: 9039; 7981	89.5%; 88.6%; 80.6%	5-year survival 75.2%; 74.9%; 67.7%
Sundaram et al. [41]	2002–2014	ACLF grade 3 : 2349	ACLF grade 3: 79.8%	NA
Marciano et al. [17]	2010–2016	34; 18; 8	82.3%; 100.0%; 62.5%	NA
Sundaram et al. [25]	2005–2016	7375; 7513; 6381	89.1%; 88.1%; 81.8%	NA
Thuluvath et al. [24]	2002–2016	4330; 3557; 3556	88%; 88%; 83%	5-year survival 74%; 74%; 70%
Huebener et al. [48]	2009–2014	24; 45; 29	3-month survival 72.4%	2-year survival : 60.2%
Artru et al. [6]	2008–2014	ACLF grade 3 : 73	ACLF grade 3: 83.6%	NA
Levesque et al. [18]	2008–2013	68; 42; 30	76.5%; 78.6%; 43.3%	NA
Michard et al. [19]	2007–2014	All grades : 55	60%	NA
Finkenstedt et al. [20]	2002–2010	All grades : 33	87%	5-year survival 82%
Xing et al. [21]	2001–2009	All grades : 133	75.9%	5-year survival 72.1%
Living-donor liver transplantation				
Kwon et al. [11]	2008–2019	261; 147; 75	ACLF grade 3: 72%	5-year survival : 67.5%
Kulkarni et al. [22]	2019–2021	All grades : 55	72.7%	NA

*Overall results across all ACLF grades if individual grade-specific data are unavailable.

Abbreviations ACLF, Acute-on-chronic liver failure; NA, not available.

In patients with ACLF, the sequential assessment of ACLF grade during the first days of management helps identify those with persistent severe ACLF, which is associated—regardless of the underlying liver disease etiology and the stage of cirrhosis prior to ACLF—with a very low probability of survival without transplantation (estimated between 10% and 20% at 28 days for ACLF grade 3) [2]. Moreover, in patients with persistent severe ACLF, most deaths occur within 15 days of admission, and their mortality risk is comparable to or even higher than that of patients awaiting transplantation for acute liver failure listed for transplantation. Therefore, these patients must be identified promptly to allow for LT as soon as possible. The first large multicenter study reporting LT outcomes in patients with multiple organ failure was published in 2013, demonstrating an acceptable one- and three-year survival rate of 74% and 62% respectively [23]. Liver transplantation outcomes in patients with severe ACLF, as defined by the EASL criteria, were not specifically studied until 2015. To date, 25 retrospective studies have been conducted in this population, with one-year survival rates exceeding 80% in the majority of series (Table 1) [6, 24–29]. Until recently, the available data were primarily derived from either single-center retrospective studies with limited sample sizes or analyses of large national registries. However, the latter were often criticized for lacking the granularity necessary to accurately identify patients with severe ACLF at the time of LT—particularly regarding the indication for mechanical ventilation (neurological vs. respiratory failure) and the absence of key variables such as the PaO_2_/FiO_2_ ratio, which is essential to assess respiratory function. As a result, the field had been anticipating robust prospective data, which became available in 2024 with the publication of the UK experience following the implementation of the ACLF-tier classification system. The results of this study confirmed initial findings with a 1-year survival rate of 81% among transplanted patients, compared to 0% in those who were listed but not transplanted [30]. In the ongoing large, prospective, international multicenter observational study “CHANCE” (Liver Transplantation in Patients With CirrHosis and Severe Acute-on-Chronic Liver Failure: iNdications and outComEs–NCT04613921), interim analyses have also confirmed this trend, reporting a 3-month mortality rate of only 9% among liver-transplanted patients with severe ACLF [31]. Notably, in large cohort studies, outcomes—while acceptable across all patients with severe ACLF—are more favorable in those with fewer organ failures. One-year survival rates are higher in patients with grade 2 ACLF compared to those with grade 3 ACLF involving three organ failures, and even lower in those with four to six organ failures. Nevertheless, even in the latter group, one-year survival has been reported to exceed 80% in the largest retrospective series [24, 25].

### Long-Term Outcomes

Few studies have assessed long-term patient and graft survival following LT in the context of severe ACLF. The most notable study, based on U.S. registry data, did not identify an increased risk of death or graft loss beyond the first year post-LT in patients transplanted with ACLF [26]. In this study, most deaths in the ACLF transplant group occurred within the first year and were primarily related to infectious complications or cardiovascular events. Long-term data from the French tricentric study published in 2025 reported no excess mortality or graft loss at 5 and 10 years in patients with ACLF grade 3 compared to matched patients with ACLF grade 1 and 2 or without ACLF. The 5-year survival rates for patients with ACLF grades 1, 2, and 3 were 73%, 71%, and 76%, respectively [32]. Notably, patients transplanted in the context of severe ACLF tend to have an increased risk of death from infectious or cardiovascular events compared to patients transplanted in other settings [32]. A recent report from King’s College Hospital confirmed favorable long-term outcomes in patients transplanted while in the ICU, with a 5-year survival rate exceeding 80% (81.9%) [30].

## Short-Term Outcomes of LDLT

Few studies have investigated survival after LT from a living donor in patients with ACLF at the time of transplantation. These studies have been conducted exclusively in Asia, are all single-center and retrospective, and involve small cohorts. Nevertheless, in these expert LDLT centers, short-term outcomes appear favorable, with survival rates even surpassing those of DDLT (Table 1) [33–36]. These findings are important and even promising, as in the context of LDLT, donor screening is rapid, allowing for timely graft allocation to the recipient. Furthermore, the transplant team can choose the optimal timing for LT based on the recipient’s clinical evolution, unlike in DDLT, where graft availability is entirely dependent on donor organ availability. This may partly explain the favorable outcomes observed with LDLT.

## Learning Curve and Selection Process

Over the past decade, LT for patients with ACLF has benefited from a significant institutional learning curve, leading to enhanced patient selection and perioperative management. A recent study based on UNOS data analyses highlighted that increasing experience with LT in critically ill patients—defined as those in the intensive care unit with one or more of the following at the time of LT: (i) grade III/IV hepatic encephalopathy, (ii) mechanical ventilation, (iii) dialysis, and/or (iv) vasopressor support—has significantly grown over the past 15 years (4.3% of total LTs in 2005–2008 vs. 7.9% in 2017–2020, *p* < 0.001) [37]. This trend has been associated with improved candidate selection: the proportion of patients transplanted while on dialysis or vasopressors increased, whereas those requiring mechanical ventilation decreased over the study period. Additionally, the waitlist time for these patients shortened [37]. These changes have translated into better post-transplant outcomes, with 1-year survival rising from 72.5% in 2005–2008 to 89.5% in 2017–2020 (*p* < 0.0001) [37]. Similarly, in a monocentric experience, an optimized pre-transplant intensive care unit management (ICU) and timely intervention has led to better post-transplant outcomes [38]. Indeed, in this study, one-year post-LT survival among patients with ACLF grade 3 at the time of transplantation increased from 66% in the 2007–2015 period to 86% in the 2015–2019 period [38]. These advancements underscore the progressive refinement of LT practices for ACLF, culminating in improved survival rates and patient care.

**FIGURE 1:**
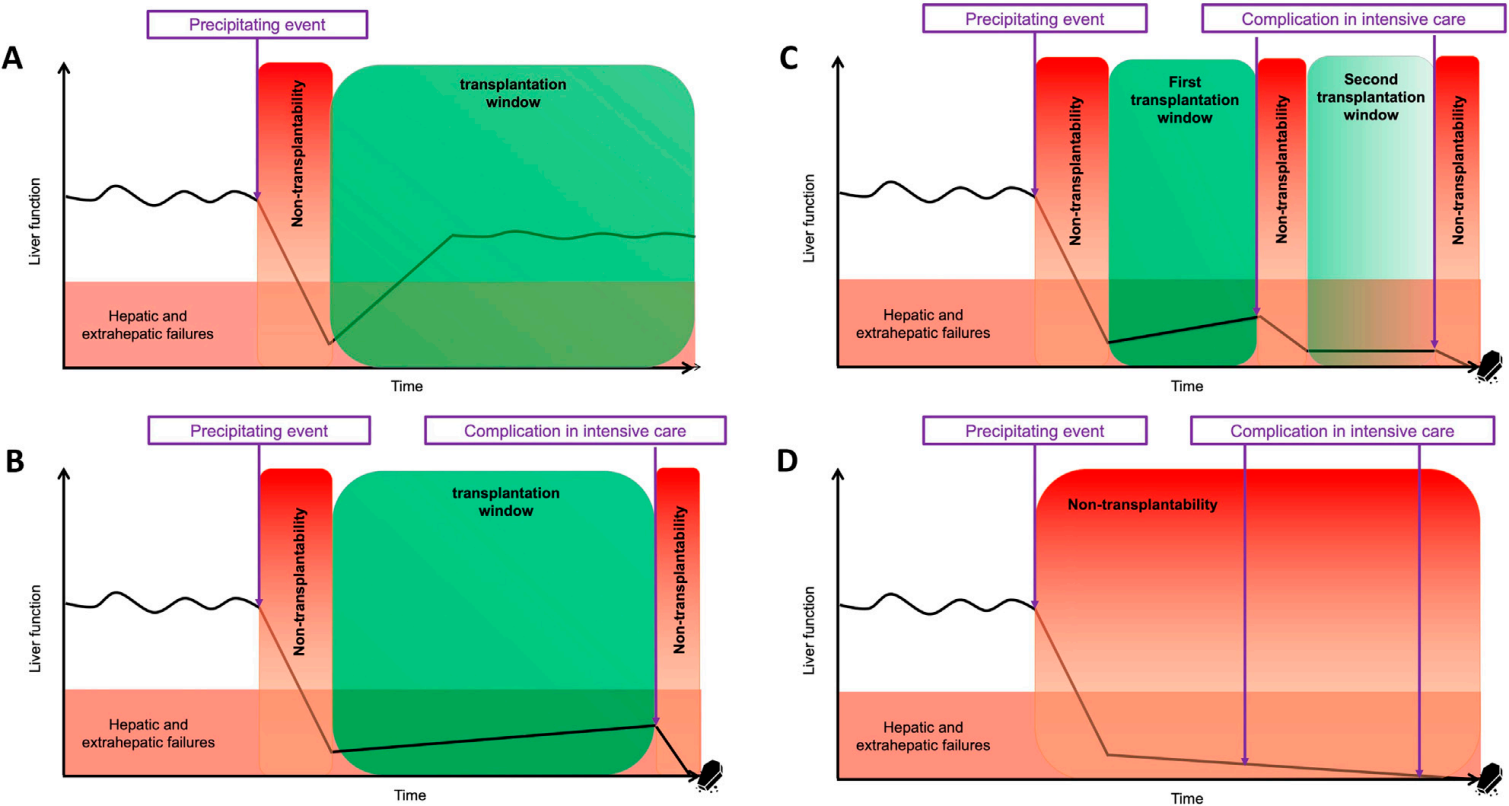
Concept of the “transplantation window” during a critical care hospitalization in severe ACLF with different scenari. **(A)** Rapid improvement of severe ACLF: patients must be referred to a LT center for evaluation as 1-year transplant-free survival is between 20% and 60%. **(B)** One transplantation window. **(C)** Two transplantation windows. The first “transplantation window” is likely more favorable (“greener”) than the second, as the patient presents with fewer complications related to hospitalization (e.g., deconditioning, colonization by multidrug-resistant bacteria, etc.). **(D)** Absence of transplantation window commonly due to uncontrolled sepsis and organ failure. *Adapted from Artru, Trovato et al., Lancet Gastroenterol. Hepatol. 2024.* [64].

While there is substantial variability across liver transplant centers in the proportion of patients with severe ACLF ultimately placed on the waiting list, estimates suggest this figure ranges between 15% and 30% of patients initially considered as potential LT candidates [39]. Approximately 50% of patients are deemed unsuitable for transplantation after the initiation of the evaluation process, primarily due to comorbidities or issues related to addiction [40]. Following placement on the waiting list, an additional 30%–40% of patients with severe ACLF die before transplantation, contributing to a highly selective process [31, 41]. As a result, the outcomes presented in Figures 1A–C reflect those of a very carefully selected subgroup of patients who ultimately underwent LT in the setting of severe ACLF.

## Predictive Factors of Mortality After Liver Transplantation for ACLF

### General Considerations

First, several patient-related factors are predictive of mortality after transplantation in the context of severe ACLF (Box 1). Among them, age stands as one of the most important factors. Indeed, in patients with severe ACLF, the risk of mortality after LT progressively increases with age beyond 50 years. At the threshold of 60 years, this risk is increased by 70%–100% [27, 42]. The presence of diabetes has also been reported as a mortality risk factor, with a 40% higher post-transplant mortality rate [26, 43]. Body mass index (BMI), as a continuous variable, also appears to be associated with an increase in post-transplant mortality [43]. Although no dedicated study has been conducted in the population of patients with severe ACLF who are candidates for transplantation, it is suggested that the presence of cirrhotic cardiomyopathy (with a prevalence of approximately 30% in this population according to the revised 2019 criteria), particularly with a septal e’ <7 cm/s, and a history of atrial fibrillation are factors associated with an increased risk of mortality after transplantation [44]. While the underlying cause of cirrhosis does not seem to impact LT outcomes, the Karnofsky index and malnutrition, assessed through sarcopenia, have both been independently associated with post-LT mortality [25, 45]. These parameters, along with frailty indices such as the Clinical Frailty Scale, which evaluate the patient’s overall condition before transplantation, play a crucial role in the transplant team’s decision-making process [46]. Finally, the presence of portal vein thrombosis, regardless of its extent and location, may also be associated with an increased risk of complications and post-transplant mortality [28].

BOX 1 Predictive factors of mortality after liver transplantation for ACLF. *Limited scientific evidence. Abbreviations: ICU, intensive care unit; PVT, portal vein thrombosis; DRI, donor risk index.

**Patient-related factors on admission to ICU**

Age (especially when ≥60 years)
Diabetes mellitus
Body mass index
Cardiac risk factors (arrhythmias, severe valvular disease, coronary artery disease)
Cumulative comorbidities as expressed in Charlson Comorbidity Index
Frailty, malnutrition – sarcopenia
PVT*
Cirrhotic cardiomyopathy*

**Factors related to a patient’s stay in ICU**

Respiratory failure as per EASL definition (PaO2/FiO2 ≤200)
Worsening organ failure, elevated arterial lactate (>4 mmol/L)
Vasopressor use and multiple vasopressors requirement
Infection with multidrug-resistant organisms during hospitalization
Prolonged time in ICU to transplantation (>15 days)*

**Donor-related factors**

High Donor Risk Index (e.g. DRI ≥1.7)
Age of the donor
Diabetes mellitus of the donor

Regarding variables dependent on the ICU stay, three key factors have been identified as robust predictors of LT outcomes (Box 1). (i) Presence of respiratory failure, defined by a PaO_2_/FiO_2_ ratio ≤200 or the need for intubation with mechanical ventilation, regardless of the underlying cause, is a major predictor of post-LT mortality [6, 25, 27, 47]. Consequently, in a patient considered for LT, successful weaning from mechanical ventilation before transplantation strongly supports proceeding with the procedure. While data on other organ failures are less robust, evidence suggests that renal replacement therapy and severe hemodynamic failure are also associated with a higher risk of post-transplant mortality. (ii) Evolution of OFs in the ICU. Stabilization or improvement of OFs before transplantation—particularly within the 48 h preceding LT—has been associated with better post-transplant prognosis. This is reflected in low arterial lactate levels on the day of LT (≤4 mmol/L) [6, 26, 48]. The presence of hemodynamic failure on the day of LT, illustrated by the use of one or two inotropes, also appears to be associated with 1-year post-LT mortality [43]. Currently, no specific norepinephrine dose threshold at the time of LT has been established in the literature beyond which transplantation would be considered unreasonable. Since they are frequently associated with acute deterioration of organ function, uncontrolled sepsis and active gastrointestinal bleeding are considered as definitive contraindications to LT [6, 46]. (iii) Time to transplantation. Favorable LT outcomes have been observed in studies where the median time from ICU admission or waitlist registration to transplantation ranges from 7 to 15 days [6, 27, 42]. Prolonged ICU stays not only increase the risk of mortality while on the waitlist but also would raise the likelihood of multidrug-resistant infections and further deterioration of the patient’s nutritional and muscular condition advocating against proceeding LT in patients with prolonged ICU stay outside of some specific cases [49].

Finally, regarding donor-dependent variables associated with mortality, the Donor Risk Index (DRI) is a reliable predictor of both patient and graft survival in the U.S., regardless of the indication for transplantation (Box 1). In the context of LT for severe ACLF, a high DRI (≥1.7) has been associated with increased short- and long-term mortality risk [25, 26]. However, these findings have not been confirmed outside the U.S., particularly in Europe, even when using a score better suited to the European graft allocation system [27, 32]. For instance, in the French retrospective tricentric study, graft characteristics did not differ between ACLF grade 3 recipients and other patients (non-ACLF and ACLF grades 1 and 2), suggesting that the favorable outcomes observed were not related to superior graft quality. In practice, the combination of a limited number of organ offers and the urgent need for transplantation imposes constraints that prevent optimal graft selection for these patients.

Potential absolute contra-indication for LT in ACLF based on a Delphi of expert in the field has been highlight in Box 2.

BOX 2 Proposed absolute contraindications to liver transplantation in the context of ACLF. *Based on Delphi consensus from Weiss et al. Transplantation 2020. [34]. #Based on the consensus document on UK ACLF Tier Bernal et al., Lancet Reg Health 2024. [18]. Abbreviations: LT, liver transplantation; ARDS, acute respiratory distress syndrome; ECMO, extra corporeal membrane oxygenation.

**Potential absolute contraindications to LT in the context of ACLF**

**Non modifiable – related to the patient**
Frailty with Clinical Frailty Scale ≥7 before admission*
**Modifiable – related to the ICU stay**
Norepinephrine requirement >1 γ/kg/min*
ARDS with PaO2/FiO2 ratio < 150*
Arterial lactate >9 mmol/L*
Active bacterial or fungal sepsis*
Severe irreversible neurological injury#
Patient under Extra Corporeal Membrane Oxygenation (ECMO) device#
Severe acute pancreatitis or intestinal ischemia#

### Concept of the Transplantation Window

The modifiable risk factors related to the ICU stay help to define a period known as the “transplantation window”. The precise definition and prospective validation of its boundaries are still lacking. These boundaries identify periods of non-transplantability surrounding the transplantation windows. However, depending on the clinical context, the number and timing of potential transplantation windows, as well as periods of non-transplantability, may vary significantly and remain currently unpredictable (Figures 1A–D). It is crucial to discuss these boundaries within the multidisciplinary team for each patient individually taking into account the non-modifiable (patients-related) risk factors and to reassess them daily to optimize access to LT and improve post-transplant outcomes. Over the past 5 years, significant efforts have been made to better define specific boundaries linked to organ failures, which help delineate the optimal transplantation window and are now integrated into the scoring systems described below.

### Scoring Systems

The UCLA-FRS (University of California Los Angeles Futility Risk Score) has been specifically developed to identify predictors of short-term mortality in patients with MELD score above 40 [44]. It incorporates the Charlson Comorbidity Index adjusted for age, the presence of septic shock prior to transplantation, and cardiovascular risk factors (including a history of arrhythmias, severe valvular disease, or significant coronary artery disease) [44].

The transplantation for ACLF-3 model (TAM) has been developed in patients with ACLF-3 at time of LT [47]. The TAM integrates age (cutoff: 53 years), respiratory failure (P/F ratio ≤200), lactatemia (cutoff: 4 mmol/L), and circulating leukocyte count (which is inversely associated with post-LT survival, with a cutoff of 10 G/L) [47]. It appears to be more predictive when calculated on the day of LT [47]. However, when recently evaluated in the French tricentric cohort, this score failed to retrospectively identify patients at risk of one-year mortality following LT [50]. Indeed, in this cohort—with a one-year survival rate of 84%—approximately 60% of patients had either an age ≥53 years or a leukocyte count ≤10 G/L, suggesting that these parameters might require further optimization.

A second specific prognostic model of patients with severe ACLF is the SALT-M score (Sundaram ACLF Liver Transplantation Mortality Score). It was published in 2023 to predict mortality after LT in patients with severe ACLF [43] and combines patient-related factors (age ≥50 years, diabetes, and body mass index) with modifiable ICU-related variables (respiratory failure and the need for vasopressors). Its predictive accuracy has been validated in an external French bicentric cohort, and it may contribute to defining the transplantation window [43].

However, while potentially useful, these scores should be considered within a comprehensive clinical approach rather than as a definitive “ultimate” decision-making tool, given their inherent uncertainty and the immediate, high-impact nature of therapeutic decisions in these critically ill patients.

## General Management in Potential Candidates to LT Admitted to ICU

Effective management of OFs is crucial to stabilizing patients with ACLF in anticipation of LT. Kidney failure, whether resulting from hepatorenal syndrome or other causes such as acute tubular injury, is the most frequently encountered organ failure in ACLF. In the intensive care setting, continuous renal replacement therapy is the preferred approach and can also aid in lowering ammonia levels in cases of severe hepatic encephalopathy as well as participating in hemodynamic stabilization [3, 51]. Circulatory failure should be addressed similarly to non-cirrhotic patients and guided by dynamic assessments, such as bedside echocardiography, to optimize fluid resuscitation and vasopressor use with norepinephrine as the first-line vasopressor and a target mean arterial pressure of 60–65 mmHg [52, 53]. Consideration of adrenal insufficiency, and cautious use of albumin (when indicated) or crystalloids are essential, while beta-blockers should be discontinued in cases of shock or renal failure [53, 54]. Patients with a Glasgow Coma Score of ≤8 require airway protection to prevent hypoxia and hypercapnia [55]. The use of benzodiazepines for sedation should be avoided. Due to the absence of specific mechanical ventilation guidelines for ACLF, management should adhere to general critical care principles, prioritizing lung-protective strategies (low tidal volumes and appropriate positive end-expiratory pressure (PEEP), particularly in ARDS patients) [53, 56]. For patients awaiting LT or receiving corticosteroids, screening for invasive fungal infection is essential; however, there are no formal recommendations for initiating empiric antifungal therapy. Any suspected bacterial infection warrants immediate and thorough investigation, followed by broad-spectrum empirical antibiotic therapy tailored to local epidemiology [57]. Antibiotic de-escalation should be considered based on clinical progression, microbiological findings, and the presence of multidrug-resistant organisms [3]. Finally, liver failure requires close monitoring of blood glucose levels and appropriate nutritional support, preferably via the enteral route. Nutritional goals should include an energy intake of 20–30 kcal/kg/day and a protein intake of 1.2–1.5 g/kg/day [3].

Furthermore, from the moment a patient with severe ACLF is admitted to ICU, the question of LT arises, along with the need for an addiction assessment in cases of alcohol-related cirrhosis. This assessment is crucial but can be challenging, particularly due to severe encephalopathy and/or orotracheal intubation. Information from the patient’s primary physician and family is essential. However, there is currently limited data in the literature to predict the risk of alcohol relapse after LT in the context of severe pre-transplant ACLF.

**FIGURE 2:**
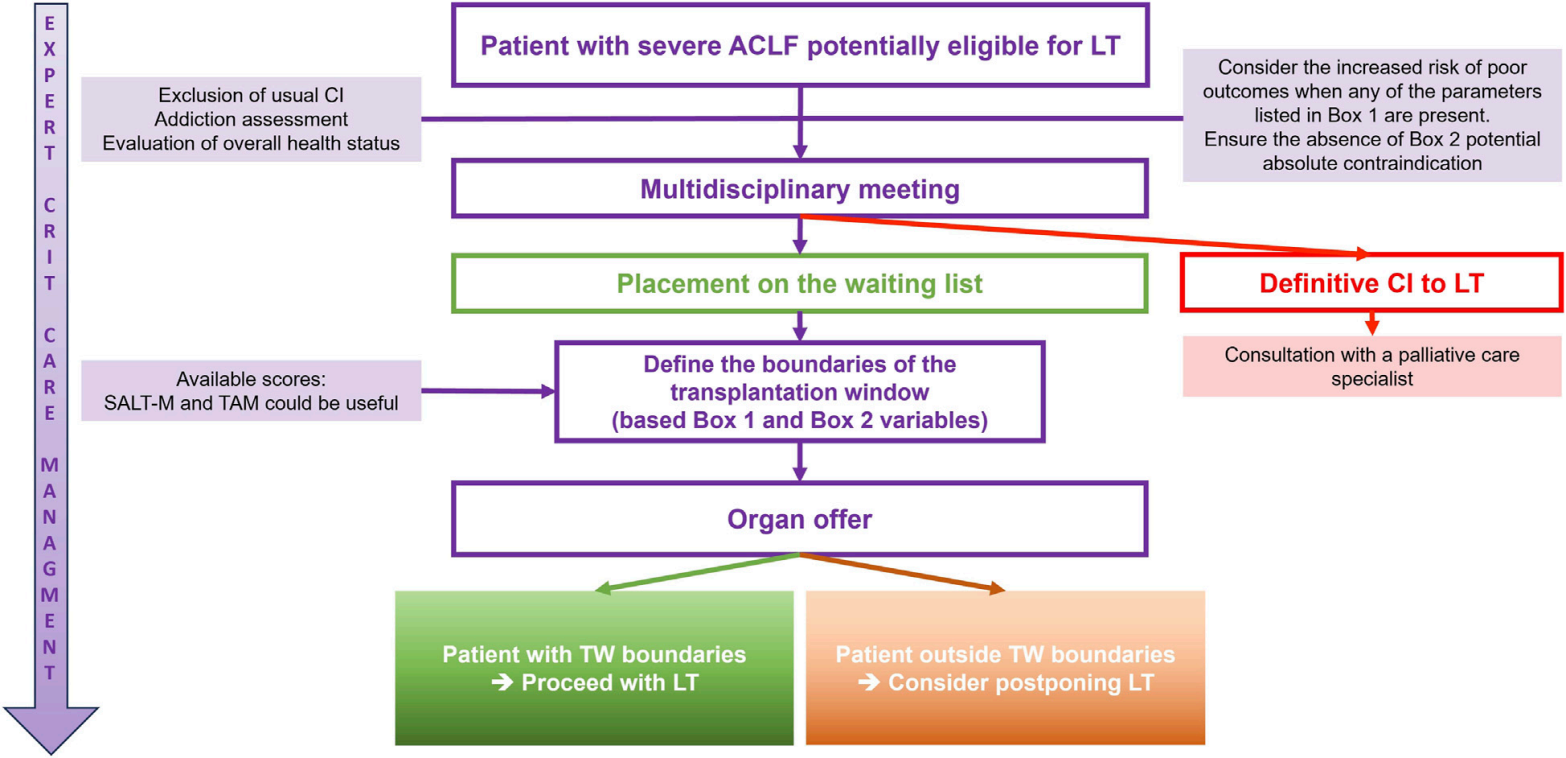
Proposal for a management algorithm for a cirrhotic patient with severe ACLF and potential eligibility for liver transplantation (LT). Abbreviations: ACLF, Acute-on-chronic liver failure; CI, contraindication; LT, liver transplantation; SALT-M, Sundaram ACLF Liver Transplantation Mortality; TAM, Transplantation for ACLF-3 model *Adapted from Artru, Trovato et al., Lancet Gastroenterol. Hepatol. 2024* [64].

Based on these considerations, a proposed algorithm for managing severe ACLF patients who may be eligible for LT is illustrated in Figure 2.

## Patient Prioritization

While OFs are the key determinant of outcomes in patients with ACLF, LT allocation policies worldwide are still largely based on MELD-derived systems, which do not account for extrahepatic organ dysfunctions. As a result, these patients are often disadvantaged compared to others with similar MELD scores but without OF outside the liver [58, 59]. These findings have been corroborated by preliminary analyses from the CHANCE study, which reported excess waitlist mortality among patients with a MELD score <30 and severe ACLF [31]. In countries where the median MELD score for access to LT is relatively low, this may be less problematic. However, in most allocation systems—especially in the context of increasing organ shortages—this may necessitate prioritization strategies to ensure graft allocation within a few days. In this regard, both Spain and the United Kingdom have taken the lead with either national or regional priority in patients with severe ACLF [30, 60]. In the UK, national priority listing can be requested for patients meeting specific high-acuity criteria. Eligible candidates include those with cirrhosis and liver failure characterized by jaundice and coagulopathy, in association with severe organ dysfunction necessitating intensive care support and an anticipated 28-day survival of less than 50%, typically corresponding to ACLF grade 3. Exclusion criteria comprise age over 60 years, comorbid conditions or ongoing alcohol use incompatible with standard LT, prior LT, active bacterial or fungal sepsis, CMV viraemia, severe irreversible neurological injury, advanced multi-organ failure with a poor trajectory, use of ECMO, significant frailty limiting rehabilitation potential, active malignancy, and severe acute pancreatitis or intestinal ischemia. In their recently published prospective studies, patients listed with national priority for ACLF received a transplant within a median of 3 days. Nevertheless, approximately 20% of candidates were not transplanted due to clinical deterioration—rates that are consistent with global reported dropout rates, even in regions with greater organ availability and in other liver transplant indications [30, 61].

Despite the growing body of evidence, ongoing debates persist regarding the inherent risks of prioritizing ACLF patients, as doing so could limit access to LT for patients with more classical indications [62]. This concern is particularly relevant in the current era, where cutting-edge research is expanding transplant eligibility to new oncological populations [63]. Although the utility of liver grafts in carefully selected oncological patients has been demonstrated, short-term outcomes for ACLF recipients—especially those transplanted from the ICU—remain inferior to those of patients with decompensated cirrhosis transplanted outside of critical care. This raises concerns about the overall impact on transplant outcomes should the indication for ACLF broaden significantly. Moreover, ACLF is a relatively common syndrome, and the potential surge in eligible candidates remains largely unexplored and difficult to predict. It is therefore crucial to identify and address the candidate-, ICU stay-, and graft-related factors contributing to poorer outcomes in this setting, with the aim of standardizing both short- and long-term outcomes. Doing so will help ensure graft utility and bridge the gap between clinical judgment and evidence-based science in candidate selection [62].

## Unmet Needs and Future Perspectives

### Organ Failure Severity and the Role of Unmeasured Variables

The limited number of studies with granular data has thus far hindered the identification of well-defined thresholds for OF severity associated with unacceptable outcomes in LT candidates with severe ACLF. For example, while the use of two vasopressors has been associated with a 3.6-fold increase in 1-year mortality [43], translating this finding into clinical practice remains challenging due to variability in vasopressor use thresholds across centers. Similarly, although a PaO_2_/FiO_2_ (P/F) ratio ≤200 is strongly linked to poor prognosis, no studies have formally investigated whether further deterioration (e.g., P/F ≤ 150) worsens outcomes—or conversely, whether improvement to a P/F ratio >250 meaningfully improves prognosis. Moreover, beyond the P/F ratio itself, it would be informative to investigate whether the specific causes of hypoxemia—such as pleural effusion, pulmonary edema, or infectious consolidation—are associated with clinical outcomes. Prospective data from the CHANCE study will certainly help address some of these critical knowledge gaps. However, given the relatively low mortality rate observed in this population, a very large sample size of transplanted patients would be required to achieve adequate statistical power. Moreover, the exhaustivity of collection of variables potentially influencing outcomes in this setting will be critical. This will be specifically the case of factors such as comorbidity scores, sarcopenia, frailty, the clinical trajectory in the final hours preceding transplantation or the experience and expertise of the transplant center. Finally, even with the collection of detailed data, it is important to note that the SALT-M score—despite being derived from a large and granular dataset—has an R^2^ value below 20%, meaning that it explains less than one-fifth of the variability in outcomes [43]. Although the CHANCE study may improve the predictive performance of existing models, it remains essential to stay cautious and acknowledge the limitations of scoring systems to predict mortality risk following LT. At this stage and given the major implications of the decision to proceed with LT, such systems should not be regarded as the final arbiter but rather serve as supportive tools to inform and guide clinical judgment.

### Pre-transplant Assessment

In patients with ACLF undergoing LT, two clinical scenarios are typically encountered. About one-third experience ACLF while already listed for transplantation, having undergone standard pre-transplant assessments [30, 39, 64]. In contrast, many patients are first considered for transplantation only after ICU admission, necessitating a rapid evaluation during ongoing critical illness—a process for which specific guidelines are currently lacking. Recent data have shown that the pre-transplant work-up in ACLF patients is often abbreviated compared to other transplant candidates, typically limited to transthoracic echocardiography, body CT, upper gastrointestinal endoscopy, and PSA testing in male patients [65]. While this streamlined work-up allows for rapid listing, it may come at the cost of reduced sensitivity in identifying contraindications—particularly cardiovascular ones—given the elevated risk of cardiovascular-related mortality in this population. The potential role of non-invasive approaches—such as coronary CT angiography or newly developed tools like the Coronary Artery Disease in Liver Transplantation (CAD-LT) score—to identify patients at highest risk for significant coronary artery disease requiring invasive angiography prior to LT warrants further investigation, particularly in this unstable population for whom transport are often challenging [66]. Finally, thorough assessment of the addiction profile warrants dedicated investigation to help standardize access to LT, especially when direct discussion is not possible due to encephalopathy and/or intubation. In particular, the relative importance of input from relatives, the general practitioner, and the addiction specialist who followed the patient prior to hospitalization appears crucial and should be formally investigated.

### Peri-Transplant Management

A major unmet need in the management of ACLF lies in the standardization of the management of the peri-transplant period, particularly as a bridge to transplantation. Extracorporeal liver support systems, such as plasma exchange (PLEX), represent a promising area of investigation. The ongoing APACHE phase 3 randomized trial (NCT03702920) is currently evaluating high-volume plasma exchange with albumin 5% (PE-A5%) in combination with standard medical therapy in patients with moderate-to-severe ACLF. Building on encouraging pilot data, APACHE is designed to assess whether PE-A5% can improve survival and organ function in these high-risk patients, with the potential to enhance transplant eligibility and post-transplant outcomes. Renal replacement therapy (RRT), often necessary in ACLF due to frequent kidney involvement, must also be integrated into a multimodal support strategy, alongside tailored nutritional support to address the severe catabolic state of these patients. The integration of Enhanced Recovery After Surgery (ERAS)-inspired bundles of care—including early mobilization, optimized hemodynamic monitoring, and protocolized organ support—could contribute to better peri-transplant conditioning and reduce postoperative complications however their applicability to this specific population remains uncertain [67]. In parallel, particular attention should be paid to mitigating cardiovascular risk during the perioperative period. While optimizing pre-transplant cardiovascular assessment is essential, early and comprehensive post-transplant evaluation should also be systematically considered. This delayed but targeted approach may help detect silent coronary artery disease and reduce the risk of early post-transplant cardiac complications. Furthermore, infection remains a leading cause of mortality in ACLF and post-transplant periods, necessitating a more personalized immunosuppression strategy. Emerging approaches based on immune functional markers, such as monocytic HLA-DR expression, may allow for dynamic risk stratification and individualized immunomodulation [68]. These strategies represent critical components in the development of a comprehensive, patient-centered peri-transplant management paradigm in ACLF.

**FIGURE 3:**
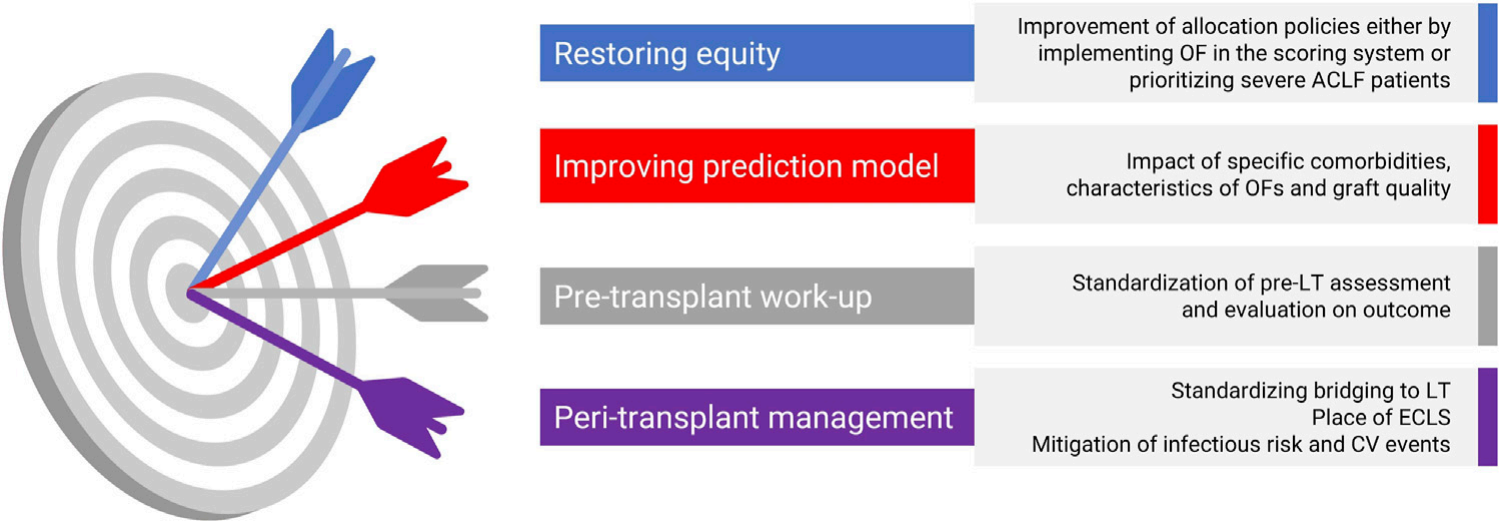
Key unmet needs and future directions in liver transplantation for ACLF. Summary of four priority areas to improve LT outcomes in ACLF: (1) restoring equity via adapted allocation policies; (2) refining prediction models by integrating comorbidities, OF profiles, and graft quality; (3) standardizing pre-LT evaluation; and (4) optimizing peri-transplant management, including bridging strategies, ECLS use, and mitigation of infectious and CV risks. Abbreviations: ACLF, acute-on-chronic liver failure; LT, liver transplantation; OF, organ failure; ECLS, extracorporeal liver support; CV, cardiovascular.

Unmet needs and future directions have been summarized in the Figure 3.

## Conclusion

LT remains a cornerstone of treatment for well-selected patients with ACLF. Accumulated data suggest that LT in ACLF leads to an acceptable survival rate at 1 year (often exceeding 80%) and at 5 years (76%). These outcomes are influenced by several factors, including the grade of ACLF, the timing of transplantation, and patient-specific characteristics such as age, diabetes, and comorbidities like cirrhotic cardiomyopathy. Predictive factors for post-transplant mortality are crucial in identifying patients who are most likely to benefit from LT, with the severity of OFs during ICU admission playing a key role.

However, many questions remain unresolved regarding the management of ACLF patients in the transplant setting. Specifically, the impact of various thresholds for OF severity on outcomes and the role of unmeasured variables, such as frailty and sarcopenia, require further exploration. Additionally, the challenge of managing patients who rapidly deteriorate and require urgent LT underscores the need for tailored pre-transplant assessment protocols and guidelines.

Further research is essential to optimize patient selection, refine prediction models, and better understand the long-term outcomes for these high-risk transplant recipients. The evolving landscape of LT in ACLF calls for a balanced approach, ensuring equitable access to liver transplants while maintaining graft utility and improving overall patient survival.

## References

[1] Arroyo V Moreau R Kamath PS Jalan R Ginès P Nevens F Acute-On-Chronic Liver Failure in Cirrhosis. Nat Rev Dis Primers (2016) 2:16041. 10.1038/nrdp.2016.41 27277335

[2] Arroyo V Moreau R Jalan R. Acute-On-Chronic Liver Failure. N Engl J Med (2020) 382(22):2137–45. 10.1056/NEJMra1914900 32459924

[3] European Association for the Study of the Liver. Electronic Address: Easloffice@easloffice.Eu, European Association for the Study of the Liver. EASL Clinical Practice Guidelines on Acute-On-Chronic Liver Failure. J Hepatol (2023) 79(2):461–91. 10.1016/j.jhep.2023.04.021 37364789

[4] Sarin SK Choudhury A Sharma MK Maiwall R Al Mahtab M Rahman S Acute-On-chronic Liver Failure: Consensus Recommendations of the Asian Pacific Association for the Study of the Liver (APASL): An Update. Hepatol Int (2019) 13(4):353–90. 10.1007/s12072-019-09946-3 31172417 PMC6728300

[5] Selva RA Lim SG Phyo WW Tun T Dan YY Lee YM Acute-On-chronic Liver Failure in a Multi-Ethnic Asian City: A Comparison of Patients Identified by Asia-Pacific Association for the Study of the Liver and European Association for the Study of the Liver Definitions. World J Hepatol (2017) 9(28):1133–40. 10.4254/wjh.v9.i28.1133 29075369 PMC5643261

[6] Artru F Louvet A Ruiz I Levesque E Labreuche J Ursic-Bedoya J Liver Transplantation in the Most Severely Ill Cirrhotic Patients: A Multicenter Study in Acute-On-Chronic Liver Failure Grade 3. J Hepatol (2017) 67(4):708–15. 10.1016/j.jhep.2017.06.009 28645736

[7] Clària J Stauber RE Coenraad MJ Moreau R Jalan R Pavesi M Systemic Inflammation in Decompensated Cirrhosis: Characterization and Role in Acute-On-Chronic Liver Failure. Hepatology (2016) 64(4):1249–64. 10.1002/hep.28740 27483394

[8] Moreau R Jalan R Gines P Pavesi M Angeli P Cordoba J Acute-On-Chronic Liver Failure Is a Distinct Syndrome that Develops in Patients with Acute Decompensation of Cirrhosis. Gastroenterology (2013) 144(7):1426–37.e14379. 10.1053/j.gastro.2013.02.042 23474284

[9] Farias AQ Curto Vilalta A Momoyo Zitelli P Pereira G Goncalves LL Torre A Genetic Ancestry, Race, and Severity of Acutely Decompensated Cirrhosis in Latin America. Gastroenterology (2023) 165(3):696–716. 10.1053/j.gastro.2023.05.033 37263305

[10] Artru F McPhail MJ. Immunopathogenesis of Acute on Chronic Liver Failure. Am J Transplant (2024) 24(5):724–32. 10.1016/j.ajt.2024.02.001 38346497

[11] Kwon H-M Kim JH Kim S-H Jun I-G Song J-G Moon D-B Benefits of Liver Transplant in Critically Ill Patients with Acute-on-Chronic Liver Failure: Implementation of an Urgent Living-Donor Program. Am J Transplant (2025) 25(1):150–63. 10.1016/j.ajt.2024.08.008 39155023

[12] Zhu C-X Yang L Zhao H Zhang Y Tu S Guo J Impact of Cirrhosis-Related Complications on Posttransplant Survival in Patients with Acute-on-Chronic Liver Failure. Hepatobiliary Pancreat Dis Int (2023) 22(1):64–71. 10.1016/j.hbpd.2022.09.004 36151023

[13] Xia L Qiao Z-Y Zhang Z-J Lv Z-C Tong H Tong Y Transplantation for EASL-CLIF and APASL Acute-on-Chronic Liver Failure (ACLF) Patients: The TEA Cohort to Evaluate Long-term Post-Transplant Outcomes. eClinicalMedicine (2022) 49:101476. 10.1016/j.eclinm.2022.101476 35747194 PMC9167862

[14] Cervantes-Alvarez E Vilatoba M Limon-de la Rosa N Mendez-Guerrero O Kershenobich D Torre A Liver Transplantation is Beneficial Regardless of Cirrhosis Stage or Acute-on-Chronic Liver Failure Grade: A Single-Center Experience. World J Gastroenterol (2022) 28(40):5881–92. 10.3748/wjg.v28.i40.5881 36353203 PMC9639654

[15] Goosmann L Buchholz A Bangert K Fuhrmann V Kluge S Lohse AW Liver Transplantation for Acute-on-Chronic Liver Failure Predicts Post-Transplant Mortality and Impaired Long-Term Quality of Life. Liver Int (2021) 41(3):574–84. 10.1111/liv.14756 34542228

[16] Agbim U Sharma A Maliakkal B Karri S Yazawa M Goldkamp W Outcomes of Liver Transplant Recipients With Acute-on-Chronic Liver Failure Based on EASL-CLIF Consortium Definition: A Single-Center Study. Transplant Direct (2020) 6(4):e544. 10.1097/TXD.0000000000000984 32309630 PMC7145003

[17] Marciano S Mauro E Giunta D Torres MC Diaz JM Bermudez C Impact of Acute-on-Chronic Liver Failure on Post-Transplant Survival and on Kidney Outcomes. Eur J Gastroenterol Hepatol (2019) 31(9):1157–64. 10.1097/MEG.0000000000001467 31385871

[18] Levesque E Winter A Noorah Z Daurès JP Landais P Feray C Impact of Acute-on-Chronic Liver Failure on 90-day Mortality Following a First Liver Transplantation. Liver Int (2017) 37(5):684–93. 10.1111/liv.13355 28052486

[19] Michard B Artzner T Lebas B Besch C Guillot M Faitot F Liver Transplantation in Critically Ill Patients: Preoperative Predictive Factors of Post-Transplant Mortality to Avoid Futility. Clin Transplant (2017) 31(12), 10.1111/ctr.1311512 28895204

[20] Finkenstedt A Nachbaur K Zoller H Joannidis M Pratschke J Graziadei IW Acute-on-Chronic Liver Failure: Excellent Outcomes After Liver Transplantation but High Mortality on the Wait List. Liver Transpl (2013) 19(8):879–86. 10.1002/lt.23678 23696006

[21] Xing T Zhong L Chen D Peng Z. Experience of Combined Liver-Kidney Transplantation for Acute-on-Chronic Liver Failure Patients with Renal Dysfunction. Transplant Proc (2013) 45(6):2307–13. 10.1016/j.transproceed.2013.02.127 23871182

[22] Kulkarni AV Reddy R Sharma M Iyengar S Rambhatla A GV P Healthcare Utilization and Outcomes of Living Donor Liver Transplantation for Patients with APASL-Defined Acute-on-Chronic Liver Failure. Hepatol Int (2023) 17(5):1233–40. 10.1007/s12072-023-10548-3 37273169 PMC10241142

[23] Karvellas CJ Lescot T Goldberg P Sharpe MD Ronco JJ Renner EL Liver Transplantation in the Critically Ill: A Multicenter Canadian Retrospective Cohort Study. Crit Care (2013) 17(1):R28. 10.1186/cc12508 23394270 PMC4056692

[24] Thuluvath PJ Thuluvath AJ Hanish S Savva Y. Liver Transplantation in Patients with Multiple Organ Failures: Feasibility and Outcomes. J Hepatol (2018) 69(5):1047–56. 10.1016/j.jhep.2018.07.007 30071241

[25] Sundaram V Jalan R Wu T Volk ML Asrani SK Klein AS Factors Associated with Survival of Patients with Severe Acute-On-Chronic Liver Failure before and after Liver Transplantation. Gastroenterology (2019) 156(5):1381–91. 10.1053/j.gastro.2018.12.007 30576643

[26] Sundaram V Mahmud N Perricone G Katarey D Wong RJ Karvellas CJ Longterm Outcomes of Patients Undergoing Liver Transplantation for Acute-On-Chronic Liver Failure. Liver Transpl (2020) 26(12):1594–602. 10.1002/lt.25831 32574423

[27] Belli LS Duvoux C Artzner T Bernal W Conti S Cortesi PA Liver Transplantation for Patients with Acute-On-Chronic Liver Failure (ACLF) in Europe: Results of the ELITA/EF-CLIF Collaborative Study (ECLIS). J Hepatol (2021) 75(3):610–22. 10.1016/j.jhep.2021.03.030 33951535

[28] Sundaram V Patel S Shetty K Lindenmeyer CC Rahimi RS Flocco G Risk Factors for Posttransplantation Mortality in Recipients with Grade 3 Acute-On-Chronic Liver Failure: Analysis of a North American Consortium. Liver Transpl (2022) 28(6):1078–89. 10.1002/lt.26408 35020260 PMC9117404

[29] Alukal JJ Li F Thuluvath PJ. Liver Transplantation within 7-Days of Listing Improves Survival in ACLF-3. Dig Dis Sci (2023) 68(8):3268–76. 10.1007/s10620-023-08011-2 37341883

[30] Bernal W Taylor R Rowe IA Chauhan A Armstrong MJ Allison MED Liver Transplantation for Critically Ill Patients with Acute on Chronic Liver Failure: A Prospective National Programme of Waitlist Prioritisation. Lancet Reg Health Eur (2024) 46:101067. 10.1016/j.lanepe.2024.101067 39529808 PMC11551510

[31] Gustot T Bernal W Fernández J Diaz JM de la Peña-Ramirez C Farias A LBO-004 Excess Waitlist Mortality and Survival Benefit of Liver Transplantation for Patients with Severe Acute-On-Chronic Liver Failure: Interim Results of the CHANCE Study. J Hepatol (2024) 80:S9–10. 10.1016/s0168-8278(24)00439-2

[32] Artru F Sacleux SC Ursic-Bedoya J Ntandja Wandji LC Lutu A L’Hermite S Long-Term Outcome Following Liver Transplantation of Patients with ACLF Grade 3. J Hepatol (2025) 82(1):62–71. 10.1016/j.jhep.2024.06.039 38981560

[33] Lin KH Liu JW Chen CL Wang SH Lin CC Liu YW Impacts of Pretransplant Infections on Clinical Outcomes of Patients with Acute-On-Chronic Liver Failure Who Received Living-Donor Liver Transplantation. PLoS One (2013) 8(9):e72893. 10.1371/journal.pone.0072893 24023787 PMC3759387

[34] Bhatti ABH Dar FS Butt MO Sahaab E Salih M Shah NH Living Donor Liver Transplantation for Acute on Chronic Liver Failure Based on EASL-CLIF Diagnostic Criteria. J Clin Exp Hepatol (2018) 8(2):136–43. 10.1016/j.jceh.2017.11.007 29892176 PMC5992305

[35] Lu CY Chen CL Ho CM Hsiao CY Wu YM Ho MC Dynamic Prognostication in Transplant Candidates with Acute-On-Chronic Liver Failure. J Pers Med (2020) 10(4):230. 10.3390/jpm10040230 33203142 PMC7711531

[36] Wang YC Yong CC Lin CC Alam H Naseer F Lin YH Excellent Outcome in Living Donor Liver Transplantation: Treating Patients with Acute-On-Chronic Liver Failure. Liver Transpl (2021) 27(11):1633–43. 10.1002/lt.26096 33977657

[37] Artzner T Goldberg DS Sundaram V Faitot F Karvellas CJ Asrani SK. Improvement in Survival after Transplantation for Critically Ill Patients with Cirrhosis in the United States. Am J Gastroenterol (2024) 120:576–83. 10.14309/ajg.0000000000002944 38976367

[38] Michard B Artzner T Deridder M Besch C Addeo P Castelain V Pretransplant Intensive Care Unit Management and Selection of Grade 3 Acute-On-Chronic Liver Failure Transplant Candidates. Liver Transpl (2022) 28(1):17–26. 10.1002/lt.26280 34431204

[39] Artzner T Bernal W Belli LS Conti S Cortesi PA Sacleux SC Location and Allocation: Inequity of Access to Liver Transplantation for Patients with Severe Acute-On-Chronic Liver Failure in Europe. Liver Transplant (2022) 28(9):1429–40. 10.1002/lt.26499 35544360

[40] Sacleux SC Ichaï P Coilly A Boudon M Lemaitre E Sobesky R Liver Transplant Selection Criteria and Outcomes in Critically Ill Patients with ACLF. JHEP Rep (2024) 6(1):100929. 10.1016/j.jhepr.2023.100929 38074503 PMC10703599

[41] Sundaram V Shah P Wong RJ Karvellas CJ Fortune BE Mahmud N Patients with Acute on Chronic Liver Failure Grade 3 Have Greater 14-Day Waitlist Mortality Than Status-1a Patients. Hepatology (2019) 70(1):334–45. 10.1002/hep.30624 30908660 PMC6597310

[42] Sundaram V Kogachi S Wong RJ Karvellas CJ Fortune BE Mahmud N Effect of the Clinical Course of Acute-On-Chronic Liver Failure Prior to Liver Transplantation on Post-Transplant Survival. J Hepatol (2020) 72(3):481–8. 10.1016/j.jhep.2019.10.013 31669304 PMC7183313

[43] Hernaez R Karvellas CJ Liu Y Sacleux SC Khemichian S Stein LL The Novel SALT-M Score Predicts 1-Year Post-transplant Mortality in Patients with Severe Acute-On-Chronic Liver Failure. J Hepatol (2023) 79(3):717–27. 10.1016/j.jhep.2023.05.028 37315809 PMC12036733

[44] Petrowsky H Rana A Kaldas FM Sharma A Hong JC Agopian VG Liver Transplantation in Highest Acuity Recipients: Identifying Factors to Avoid Futility. Ann Surg (2014) 259(6):1186–94. 10.1097/SLA.0000000000000265 24263317

[45] Artru F le Goffic C Pageaux GP Saliba F Louvet A. Sarcopenia Should Be Evaluated in Patients with Acute-On-Chronic Liver Failure and Candidates for Liver Transplantation. J Hepatol (2022) 76(4):983–5. 10.1016/j.jhep.2021.09.004 34536432

[46] Weiss E Saner F Asrani S Biancofiore G Blasi A Lerut J When Is a Critically Ill Cirrhotic Patient Too Sick to Transplant? Development of Consensus Criteria by a Multidisciplinary Panel of 35 International Experts. Transplantation (2020) 105:561–8. 10.1097/TP.0000000000003364 32568955

[47] Artzner T Michard B Weiss E Barbier L Noorah Z Merle JC Liver Transplantation for Critically Ill Cirrhotic Patients: Stratifying Utility Based on Pretransplant Factors. Am J Transpl (2020) 20(9):2437–48. 10.1111/ajt.15852 32185866

[48] Huebener P Sterneck MR Bangert K Drolz A Lohse AW Kluge S Stabilisation of Acute-On-Chronic Liver Failure Patients before Liver Transplantation Predicts Post-Transplant Survival. Aliment Pharmacol Ther (2018) 47(11):1502–10. 10.1111/apt.14627 29611203

[49] Fernández J Prado V Trebicka J Amoros A Gustot T Wiest R Multidrug-Resistant Bacterial Infections in Patients with Decompensated Cirrhosis and with Acute-On-Chronic Liver Failure in Europe. J Hepatol (2019) 70(3):398–411. 10.1016/j.jhep.2018.10.027 30391380

[50] Artru F Sacleux SC Ursic-Bedoya J Pageaux GP Louvet A Saliba F. Evaluation of the Transplantation of ACLF Grade 3 Model (TAM) in the Multicenter French Experience. Liver Transpl (2023) 29(7):785–7. 10.1097/LVT.0000000000000111 36847134

[51] Perricone G Artzner T De Martin E Jalan R Wendon J Carbone M. Intensive Care Management of Acute-On-Chronic Liver Failure. Intensive Care Med (2023) 49(8):903–21. 10.1007/s00134-023-07149-x 37552333

[52] De Backer D Biston P Devriendt J Madl C Chochrad D Aldecoa C Comparison of Dopamine and Norepinephrine in the Treatment of Shock. N Engl J Med (2010) 362(9):779–89. 10.1056/NEJMoa0907118 20200382

[53] Karvellas CJ Gustot T Fernandez J. Management of the Acute on Chronic Liver Failure in the Intensive Care Unit. Liver Int (2023) 45:e15659. 10.1111/liv.15659 37365997 PMC11815614

[54] Tsai MH Peng YS Chen YC Liu NJ Ho YP Fang JT Adrenal Insufficiency in Patients with Cirrhosis, Severe Sepsis and Septic Shock. Hepatology (2006) 43(4):673–81. 10.1002/hep.21101 16557538

[55] Nadim MK Durand F Kellum JA Levitsky J O’Leary JG Karvellas CJ Management of the Critically Ill Patient with Cirrhosis: A Multidisciplinary Perspective. J Hepatol (2016) 64(3):717–35. 10.1016/j.jhep.2015.10.019 26519602

[56] Acute Respiratory Distress Syndrome Network, Brower RG Matthay MA Morris A Schoenfeld D Thompson BT Ventilation with Lower Tidal Volumes as Compared with Traditional Tidal Volumes for Acute Lung Injury and the Acute Respiratory Distress Syndrome. N Engl J Med (2000) 342(18):1301–8. 10.1056/NEJM200005043421801 10793162

[57] Evans L Rhodes A Alhazzani W Antonelli M Coopersmith CM French C Surviving Sepsis Campaign: International Guidelines for Management of Sepsis and Septic Shock 2021. Intensive Care Med (2021) 47(11):1181–247. 10.1007/s00134-021-06506-y 34599691 PMC8486643

[58] Sundaram V Shah P Mahmud N Lindenmeyer CC Klein AS Wong RJ Patients with Severe Acute-On-Chronic Liver Failure Are Disadvantaged by Model for End-Stage Liver Disease-Based Organ Allocation Policy. Aliment Pharmacol Ther (2020) 52(7):1204–13. 10.1111/apt.15988 32725664

[59] Hernaez R Liu Y Kramer JR Rana A El-Serag HB Kanwal F. Model for End-Stage Liver Disease-Sodium Underestimates 90-Day Mortality Risk in Patients with Acute-On-Chronic Liver Failure. J Hepatol (2020) 73(6):1425–33. 10.1016/j.jhep.2020.06.005 32531416 PMC10424237

[60] Rodríguez-Perálvarez M Gómez-Bravo MÁ Sánchez-Antolín G De la Rosa G Bilbao I Colmenero J Expanding Indications of Liver Transplantation in Spain: Consensus Statement and Recommendations by the Spanish Society of Liver Transplantation. Transplantation (2021) 105(3):602–7. 10.1097/TP.0000000000003281 32345868

[61] Tanaka T Wehby G Vander Weg M Mueller K Axelrod D. US Population Size and Outcomes of Adults on Liver Transplant Waiting Lists. JAMA Netw Open (2025) 8(3):e251759. 10.1001/jamanetworkopen.2025.1759 40131274 PMC11937946

[62] Artru F Goldberg D Kamath PS. Should Patients with Acute-On-Chronic Liver Failure Grade 3 Receive Higher Priority for Liver Transplantation? J Hepatol (2023) 78(6):1118–23. 10.1016/j.jhep.2022.12.026 37208098

[63] Adam R Piedvache C Chiche L Adam JP Salamé E Bucur P Liver Transplantation Plus Chemotherapy versus Chemotherapy Alone in Patients with Permanently Unresectable Colorectal Liver Metastases (TransMet): Results from a Multicentre, Open-Label, Prospective, Randomised Controlled Trial. Lancet (2024) 404(10458):1107–18. 10.1016/S0140-6736(24)01595-2 39306468

[64] Artru F Trovato F Morrison M Bernal W McPhail M. Liver Transplantation for Acute-On-Chronic Liver Failure. Lancet Gastroenterol Hepatol (2024) 9(6):564–76. 10.1016/S2468-1253(23)00363-1 38309288

[65] Bellec C Moirand R Abergel A Antonini T Anty R Mabile-Archambeaud I THU-079 Pre-Transplant Work-Up for Acute-On-Chronic Liver Failure Patients in Intensive Care Units, a Nationwide French Survey. J Hepatol (2024) 80:S168. 10.1016/s0168-8278(24)00762-1

[66] Rachwan RJ Kutkut I Timsina LR Bou Chaaya RG El-Am EA Sabra M CAD-LT Score Effectively Predicts Risk of Significant Coronary Artery Disease in Liver Transplant Candidates. J Hepatol (2021) 75(1):142–9. 10.1016/j.jhep.2021.01.008 33476745

[67] Noba L Rodgers S Chandler C Balfour A Hariharan D Yip VS. Enhanced Recovery after Surgery (ERAS) Reduces Hospital Costs and Improve Clinical Outcomes in Liver Surgery: A Systematic Review and Meta-Analysis. J Gastrointest Surg (2020) 24(4):918–32. 10.1007/s11605-019-04499-0 31900738 PMC7165160

[68] Delignette MC Riff A Antonini T Soustre T Bodinier M Peronnet E Individual mHLA-DR Trajectories in the ICU as Predictors of Early Infections Following Liver Transplantation: A Prospective Observational Study. Crit Care (2025) 29(1):79. 10.1186/s13054-025-05305-x 39966934 PMC11834174

